# Perioperative Chemotherapy in Poorly Differentiated Neuroendocrine Neoplasia of the Bladder: A Multicenter Analysis

**DOI:** 10.3390/jcm9051351

**Published:** 2020-05-05

**Authors:** Giuseppe Lamberti, Maria Pia Brizzi, Sara Pusceddu, Fabio Gelsomino, Giovanni Di Meglio, Francesco Massari, Giuseppe Badalamenti, Ferdinando Riccardi, Toni Ibrahim, Chiara Ciccarese, Sebastiano Buti, Carlo Carnaghi, Natalie Prinzi, Francesco Panzuto, Davide Campana

**Affiliations:** 1 Department of Experimental, Diagnostic and Specialty Medicine, S.Orsola-Malpighi University Hospital, 40138 Bologna, Italy; lamberti.giu88@gmail.com; 2 AOU S.Luigi Gonzaga, 10043 Orbassano (Torino), Italy; brizzimariapia@gmail.com; 3 Department of Medical Oncology, Fondazione IRCCS Istituto Tumori Milano, ENETS Center of Excellence, 20133 Milan, Italy; sara.pusceddu@istitutotumori.mi.it (S.P.); natalie.prinzi@istitutotumori.mi.it (N.P.); 4 Department of Oncology and Hematology, University Hospital of Modena, 41125 Modena, Italy; fabiogelsomino83@yahoo.it; 5 Division of Medical Oncology, Ospedale Centrale di Bolzano, 39100 Bolzano, Italy; giovannidimeglio@hotmail.it (G.D.M.); carlo.carnaghi@sabes.it (C.C.); 6 Division of Oncology, S.Orsola-Malpighi Hospital, 40138 Bologna, Italy; francesco.massari@aosp.bo.it; 7 Medical Oncology, Department of Surgical, Oncological and Stomatological Sciences, University of Palermo, 90127 Palermo, Italy; Giuseppe.badalamenti@unipa.it; 8 Oncologia - AORN Cardarelli, 80131 Naples, Italy; nando.riccardi@gmail.com; 9 Osteoncology and Rare Tumors Center- Istituto Scientifico Romagnolo per lo Studio e la Cura dei Tumori (IRST) IRCSS, 47014 Meldola (Forlì-Cesena), Italy; toni.ibrahim@irst.emr.it; 10 Medical Oncology, Azienda Ospedaliera Universitaria Integrata, University of Verona, 37126 Verona, Italy; ciccarese.c@gmail.com; 11 Fondazione Policlinico «A. Gemelli» IRCCS, 00168 Roma, Italy; 12 Medical Oncology Unit, University Hospital of Parma, 43126 Parma, Italy; sebabuti@libero.it; 13 Digestive Disease Unit, Sant’Andrea University Hospital, ENETS Center of Excellence, 00189 Rome, Italy; fpanzuto@ospedalesantandrea.it; 14 Department of Medical and Surgical Sciences, S.Orsola-Malpighi University Hospital, 40138 Bologna, Italy

**Keywords:** neuroendocrine carcinoma (NEC), perioperative chemotherapy, bladder, disease-specific survival, prognostic factors, small cell carcinoma

## Abstract

There is scant evidence about optimal management of poorly differentiated neuroendocrine carcinoma of the bladder (BNEC). We performed a multicenter retrospective study on BNEC patients from 13 Italian neuroendocrine-dedicated centers to analyze strategies associated with better outcomes. Mixed adeno-neuroendocrine carcinomas (MANEC) were included. We analyzed overall survival (OS) in the overall cohort, relapse-free survival (RFS) in radically operated patients and progression-free survival (PFS) in patients who received chemotherapy for metastatic disease. Fifty-one BNEC patients were included (male: 46, median age: 70 years). Overall, median OS was 16.0 months, radical tumor resection was performed in 37 patients (72.5%) and 11 of these (29.7%) also received peri-operative platinum-etoposide chemotherapy. Median OS was longer in patients with better performance status (PS) and in those with stage I–III disease at diagnosis compared to stage IV. Among patients who underwent radical tumor resection (*N* = 37), RFS was longer in patients with better PS and showed a trend towards a longer RFS in those treated with peri-operative chemotherapy than with surgery alone (11 vs. 6 months; *p* = 0.078). Among 28 patients receiving chemotherapy for metastatic disease, PFS was 5.0 months and there was a trend towards improved PFS in patients receiving carboplatin-etoposide chemotherapy compared to other regimens. A multivariate model unmasked a significant association between carboplatin-etoposide chemotherapy and risk for disease progression or death (HR: 0.39 (95%CI: 0.16–0.96) *p* = 0.040). Performance status might be associated with improved RFS in radically operated patients, while type of chemotherapy might affect PFS in patients receiving chemotherapy for metastatic BNEC.

## 1. Introduction

Poorly differentiated neuroendocrine carcinoma (NEC) of the bladder (BNEC) is a rare type of bladder cancer, summing up to less than 1% of bladder malignancies [1]. The neuroendocrine component can be found either alone, mostly with small-cell or large-cell morphology, or can be mixed to an epithelial component (mixed adeno-neuroendocrine carcinoma, MANEC), that is usually a urothelial carcinoma [1]. Some features of BNEC are shared with small-cell carcinoma of the lung, such as pathology [2] and the typical genomic alterations, TP53 and RB1 mutation or loss [3]. On the clinical side, BNEC is associated with smoking habits and has dismal prognosis, with five-year survival rates from diagnosis ranging from 10.5% to 16% [4,5].

Resectable BNEC may be treated with surgery (cystectomy or bladder sparing) with or without peri-operative chemotherapy (either adjuvant or neo-adjuvant), or radiation therapy. Although neo-adjuvant chemotherapy is the recommended strategy for resectable BNEC, there is no prospective trial comparing different treatment strategies because of the rarity of the disease, so treatment modalities usually mirror those used in small-cell lung cancer. In keeping with this, treatment of metastatic disease with platinum-etoposide chemotherapy mirrors small-cell lung cancer treatment recommendations. However, clinical course and best treatment strategy are far from being clear.

We led a multicenter retrospective study among European neuroendocrine tumor society (ENETS) centers of excellence in Italy and sought to describe clinical behavior and clinicopathological features that affect outcomes of BNEC patients.

## 2. Experimental Section

### 2.1. Study Design

A retrospective analysis of a prospectively maintained database was performed. Data about patients with BNEC were requested from 13 Italian centers coordinated by It.a.net, the Italian association for neuroendocrine tumors. According to ENETS requirements, data were prospectively collected for all patients with neuroendocrine neoplasia at each center and then retrospectively aggregated in a common electronic database. We performed a retrospective analysis on all consecutive patients treated for a BNEC at the participating institutions. Inclusion criteria were ≥18 years of age, histologically confirmed diagnosis of BNEC, available stage at diagnosis and at least one follow-up visit after diagnosis. MANEC were included, while paragangliomas were excluded [6].

Data about sex, date of birth, date of diagnosis, age at diagnosis, presence of cancer-related symptoms, performance status according to Eastern Cooperative Oncology Group (ECOG PS) classification, World Health Organization (WHO) 2010 classification [7], stage at diagnosis according to the 8th American Joint Cancer Committee TNM staging system [8], treatment received (including surgery, type of surgery, chemotherapy, timing and type of chemotherapy), follow-up and survival status at last follow-up were collected. All pathology reports were performed by a pathologist expert in neuroendocrine neoplasia (NEN) at each center. Patients with metastatic disease at first radiological evaluation after surgical resection were considered metastatic at diagnosis. Chemotherapy administered within three months before surgery was categorized as neoadjuvant chemotherapy, while chemotherapy delivered within three months after surgery in patients with no macroscopical residual disease (localized or distant) was categorized as adjuvant chemotherapy. Perioperative chemotherapy was considered as having received either neoadjuvant or adjuvant chemotherapy. Follow-up was performed by means of computed tomography scan or magnetic resonance of the abdomen every three months (+/− one month) according to local practice.

All patients or their legal representatives provided written informed consent for anonymous review of their data for research purposes. The study protocol was approved by the local Institution Review Board (Comitato Etico Indipendente, S.Orsola-Malpighi University Hospital, Bologna) and was conducted in accordance with the Declaration of Helsinki (6th revision, 2008).

### 2.2. Statistical Analysis

Descriptive statistics were reported as frequency, mean and range or median and interquartile range (IQR, 25th–75th percentile) as appropriate. The primary endpoint of the study in the overall cohort was overall survival (OS), defined as the time from diagnosis to death for any cause or last follow-up date. In the cohort of patients treated with curative intent, the primary endpoint was relapse-free survival (RFS), defined as the time from surgery to radiological evidence of disease relapse or death, whichever occurred first, or last radiological assessment with no evidence of disease relapse. In this cohort OS was also calculated. In the cohort of patients who received chemotherapy for metastatic disease, we defined progression-free survival (PFS) as the time from chemotherapy start to radiological progression per investigator assessment or death for any cause, whichever occurred first, or last radiological assessment time; in this cohort, we also evaluated the survival time between chemotherapy start and death from any cause or last follow-up time. Survival time was estimated using the Kaplan–Meier method and results were compared using the log-rank test. Risk factor for survival was evaluated with univariate and multivariate analysis by Cox proportional hazard method. Multivariate analysis was performed using the backward stepwise method after including all variables with significant or near-significant association (*p* ≤ 0.10) at either log-rank or univariate analysis by Cox proportional hazard method. Risk factors were reported with their hazard ratio (HR) and its respective 95% confidence interval (95% CI). All performed analyses were summarized and reported in tables and figures. All *p*-values were two-tailed and a *p*-value < 0.05 was considered significant. All statistical analyses were performed using dedicated software (IBM—SPSS Statistics version 22.0, SPSS Inc., Chicago, IL, USA, and R Statistical package version 3.6.1, R Foundation for Statistical Computing, Vienna, Austria).

## 3. Results

### 3.1. Study Population

Data about 72 patients with bladder NENs were collected at the participating institutions between 2005 and 2018. Four patients with bladder paraganglioma, 12 with incomplete diagnosis data and five with no follow-up data after diagnosis were excluded, resulting in 51 BNEC patients included in the final analysis population. A summary of patient characteristics is reported in Table 1.

Forty-six patients (46/51, 90.2%) were male and median age at diagnosis was 70 years (range 47–85). At diagnosis, 38 patients (38/42, 90.5%) had cancer-related symptoms (hematuria in 28, irritative voiding symptoms in six, pain in three, fatigue and weight loss in one), while ECOG PS was zero in 22 patients (22/48, 45.8%) and 1–2 in 26 patients (26/48, 54.2%). Histology was NEC in 41 patients (41/51, 80.4%) and MANEC in 10 (10/51, 19.6%), and all of them had a urothelial carcinoma component. At diagnosis, BNEC was potentially resectable (stage I–III) in 38 patients (38/51, 74.5%) and metastatic (stage IV) in 13 patients (13/51, 25.5%). Of these, 13 (13/13, 100%) patients had liver metastases, two (2/13, 15.4%) had bone metastases, and one (1/13, 7.7%) had lung metastases.

Resection of primary lesion was performed in 46 patients (46/51, 90.2%), 40 of which underwent radical cystectomy (40/46, 78.4%). Surgery resulted in no residual disease (R0 resection) in 37 patients, 11 of which (11/37, 29.7%) received peri-operative chemotherapy (neo-adjuvant treatment in four and adjuvant treatment in seven), while 26 (26/37, 70.3%) were treated with surgery alone. All peri-operative chemotherapy regimens were platinum-based doublets with etoposide. Among the 37 patients who achieved a R0 resection, disease relapsed in 26 (26/37, 70.3%). Overall, first-line chemotherapy was administered in 28 patients (28/51, 54.9%): 19 patients (19/28, 67.9%) received platinum-based doublets chemotherapy and nine received other regimens (9/28, 32.1%).

### 3.2. Survival Analysis in the Overall Cohort

**Figure 1:**
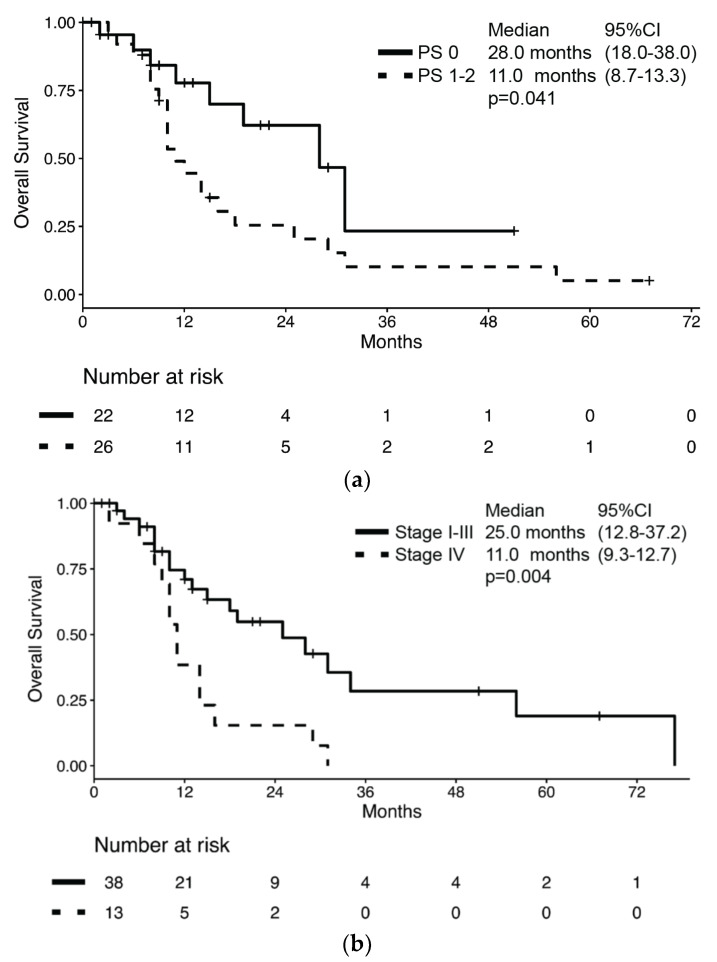
Kaplan–Meier estimates of overall survival (OS) by (**a**) performance status according to ECOG and (**b**) stage at diagnosis.

During a median follow-up of 29.0 months (95% CI 9.9–48.1), 32 patients had died for a median OS of 16.0 months (95% CI: 10.3–21.7, Supplementary Figure S1). Median OS was significantly longer in patients with PS 0 compared to those with poorer PS (28.0 months (95%CI: 18.0–38.0) vs. 11.0 months (95% CI: 8.7–13.3), respectively; *p* = 0.041, Figure 1a) and in those with stage I–III compared to those with stage IV at diagnosis (25.0 months (95% CI: 12.8–37.2) vs. 11.0 months (95% CI: 9.3–12.7), respectively; *p* = 0.004, Figure 1b).

However, no difference in median OS was observed according to sex (male: 15 months vs. female: 29 months; *p* = 0.980), presence of cancer-related symptoms (absence: 14 months vs. presence: 14 months; *p* = 0.520), histology (MANEC: 13 months vs. NEC: 16 months; *p* = 0.634) or surgery of primary lesion (any surgery of primary lesion: 18 months vs. no surgery: 14 months; *p* = 0.323). According to the univariate analysis, the only significant risk factor for death was stage at diagnosis (HR: 2.79 (95% CI: 1.33–5.85); *p* = 0.007), while no significant association between survival and sex, age, presence of cancer-related symptoms, ECOG PS, histology subtype, Ki67, and resection of primary lesion was observed (Table 2). In a multivariate model which included both ECOG PS and stage at diagnosis, only the latter retained its association with the risk of death (HR: 2.52 (95% CI: 1.19–5.35); *p* = 0.016).

### 3.3. Relapse-Free Survival Analysis in the Cohort of Patients Treated with Curative Intent

**Figure 2:**
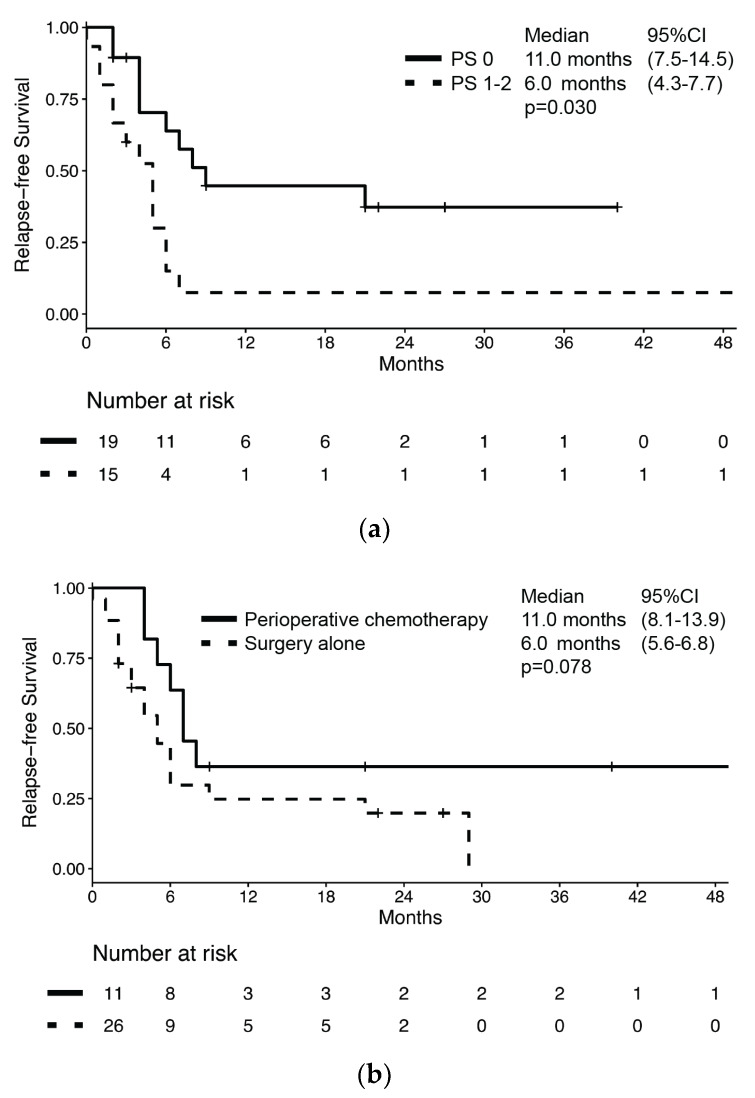
Kaplan–Meier estimates of relapse-free survival (RFS) in bladder NEC patients who underwent radical surgery of primary lesion by (**a**) performance status according to ECOG and (**b**) treatment with peri-operative chemotherapy.

Characteristics of the 37 patients who received upfront treatment with curative intent are summarized in Supplementary Table S1. Of these, BNEC relapsed in 26 patients (70.3%) for a median RFS of 7.0 months (95% CI: 4.6–9.4) and a median OS of 28.0 months (95% CI: 12.7–43.3) (Supplementary Figure S2). RFS was significantly longer in patients with PS 0 compared to those with poorer PS (11.0 months (95% CI: 7.5–14.5) vs. 6.0 months (95% CI: 4.3–7.7), respectively; *p* = 0.030, Figure 2a). The RFS difference by perioperative chemotherapy administration did not reach statistical significance despite the RFS being numerically longer in patients who received perioperative chemotherapy compared to those receiving surgery alone (11 months (95% CI: 8.1–13.9) vs. 6 months (95% CI: 5.2–6.8), respectively; *p* = 0.078, Figure 2b).

Nevertheless, no difference in median RFS was observed according to sex (male: 7 months vs. female: 9 months; *p* = 0.793), presence of cancer-related symptoms (absence: 16 months vs. presence: 6 months; *p* = 0.763), histology (MANEC: 4 months vs. NEC: 9 months; *p* = 0.144), or presence of nodal involvement (negative nodes: 6 months vs. positive nodes: 6 months; *p* = 0.280). According to univariate analysis, the only significant risk factor for disease relapse or death was ECOG PS (HR: 2.40 (95% CI: 1.04–5.54); *p* = 0.041), while no significant association between survival and sex, age, presence of cancer-related symptoms, nodal involvement, Ki67, and perioperative chemotherapy was observed (Table 3). In a multivariate model which included both ECOG PS and perioperative chemotherapy, only ECOG PS retained its association with the risk of disease relapse or death (HR: 2.73 (95% CI: 1.16–6.44); *p* = 0.021).

### 3.4. Survival Analysis in the Cohort of Patients who Received Chemotherapy for Metastatic Disease

**Figure 3:**
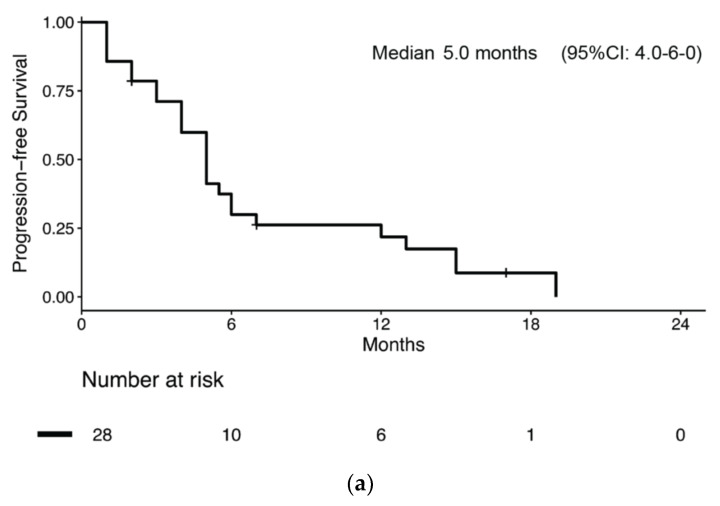

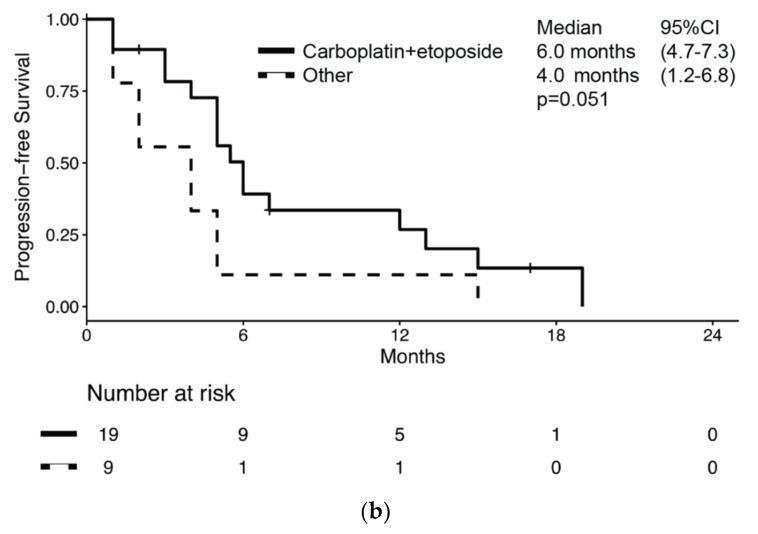
Kaplan–Meier estimates of progression-free survival (PFS) in bladder NEC patients who received chemotherapy for metastatic disease (**a**) overall and (**b**) by chemotherapy regimen received.

Overall, 28 patients (28/51, 54.9%) received chemotherapy for metastatic BNEC (Supplementary Table S2). Of these, 15 had disease relapse after previous radical surgery, while 12 had metastatic disease at diagnosis. One patient started chemotherapy for inoperable stage III BNEC. Median PFS and survival from chemotherapy start was 5.0 months (95% CI: 4.0–6.0) (Figure 3a) and 8.0 months (95% CI: 7.1–8.9), respectively. Median PFS was numerically longer in patients receiving carboplatin-etoposide chemotherapy compared to patients receiving other regimens, but without reaching statistical significance (6.0 months (95% CI: 4.7–7.3) vs. 4.0 months (95% CI: 1.2–6.8); *p* = 0.051, Figure 3b).

Moreover, no significant difference in PFS according to sex, ECOG PS, histology, stage at diagnosis, previous radical surgery was observed. At univariate analysis, the risk of progression or death was associated with carboplatin-etoposide chemotherapy (HR: 0.47 (95%CI: 0.20–1.08); *p* = 0.076) and age at diagnosis (HR: 1.06 (95%CI: 0.99–1.11); *p* = 0.063), both without reaching statistical significance (Table 4). Nevertheless, a multivariate model that corrected for both chemotherapy regimen and age showed that the risk for progression or death was associated with both carboplatin-etoposide chemotherapy (HR: 0.39 (95%CI: 0.16–0.96); *p* = 0.04) and age at diagnosis (HR: 1.06 (95%CI: 1.00–1.11); *p* = 0.036).

## 4. Discussion

In this multicenter Italian analysis, we analyzed the outcome of BNEC patients and observed a median OS of 16 months across all stages. Survival was as short as 8.0 months from chemotherapy start in those receiving chemotherapy for metastatic disease, but was up to 28.0 months in the subset of patients who underwent radical resection.

BNEC patients have poor prognosis, similar to that of small-cell and large-cell lung cancer patients, with a reported median survival slightly longer than one year across all stages, similar to our population [9,10]. However, survival is highly variable and some patients can achieve long-term remission after resection, or even a cure in those with earlier disease stages [5]. To improve survival, guidelines recommend neoadjuvant chemotherapy in resectable BNEC [11]. However, given the rarity of BNEC, this recommendation is based on scant evidence. The most recent retrospective series comprised 68 patients who underwent cystectomy for a BNEC and found that adjuvant chemotherapy was associated with improved survival [12]. In this study, one patient received neoadjuvant chemotherapy but was excluded from the analysis. In the MD Anderson Cancer Centre experience, among 95 BNEC patients who underwent cystectomy, 48 received neoadjuvant chemotherapy [13]. This approach was associated with improved overall survival and DSS after correction for confounding factors. In addition, 21 patients who did not receive prior neoadjuvant treatment received adjuvant chemotherapy. Overall survival in these patients was similar compared to those receiving cystectomy alone, and worse than survival of patients receiving neoadjuvant chemotherapy. In respect to the type of chemotherapy, cancer registries do not report this piece of information [14], while retrospective series usually include several different regimens, although a platinum-based regimen is the most adopted in the neoadjuvant setting [13], thus preventing any meaningful comparison or conclusion to be drawn. This retrospective data on which guidelines rely are difficult to interpret in light of the possibility of selection bias since neoadjuvant chemotherapy may be offered to healthier younger patients who may have better prognosis irrespective of treatment. In this regard, there is only one phase II prospective trial available in which 18 patients with resectable BNEC received neoadjuvant chemotherapy with four cycles of alternating ifosfamide/doxorubicin and cisplatin/etoposide [9]. Although median OS in patients with resectable BNEC was 58 months, the proposed regimen was cumbersome and yielded a high grade 3-4 toxicity rate (up to 37%). Moreover, the study lacked a formal statistical plan so that this approach is not widely adopted. Registry-based studies also suggest a survival advantage for BNEC patients receiving peri-operative chemotherapy compared to those who do not receive it [15,16,17]. Nevertheless, these studies suffer from limitations that are typical of registry studies, such as selection bias, lack of data verification and missing data.

We analyzed data from 51 BNEC patients of which 37 underwent radical primary lesion resection. In these patients, RFS was 7.0 months and was longer in patients with better ECOG PS. Interestingly, the administration of perioperative chemotherapy (either neoadjuvant or adjuvant platinum-based doublet with etoposide) showed a non-significant trend towards an improved RFS despite a numerically longer median RFS in patients receiving perioperative chemotherapy compared to those treated with surgery alone (11.0 vs. 6.0 months, respectively), and non-overlapping 95% CIs between the two groups as shown in Figure 2b. However, the multivariate analysis showed that only ECOG PS retained its association with RFS, which might be in keeping with the selection bias because of which healthier patients receive more intensive treatments. According to guideline recommendations, no patient received radiation therapy either with chemotherapy or after surgery in our series. In fact, there is scant evidence that radiation therapy might improve survival and it mainly comes from registry-based studies [18,19]; hence the role of peri-operative radiation therapy is still unclear. Among the 28 patients receiving chemotherapy for advanced disease, there was also a near-significant trend towards a longer median PFS in those receiving carboplatin-etoposide chemotherapy compared to those receiving other kinds of chemotherapy (6.0 vs. 4.0 months, respectively, *p* = 0.051). Notably, a multivariate model which included both age at diagnosis and type of chemotherapy unmasked a significant association between both covariates and PFS (HR: 0.39).

Our study has some limitations. First, its retrospective nature can introduce a selection bias as well. However, according to ENETS Center of Excellence requirements, data are prospectively collected in a database for each center and are gathered and analyzed retrospectively. Second, the sample size was relatively small for some subgroups and this could have affected our ability to highlight some differences (e.g., by sex). Nevertheless, it should be highlighted that BNEC is a very rare disease, accounting for less than 1% of bladder tumors.

## 5. Conclusions

In conclusion, definitive evidence is lacking regarding the optimal management of BNEC. Our study suggests that ECOG PS is associated with RFS in radically operated patients, while both age and carboplatin-etoposide chemotherapy are associated with improved survival in patients receiving chemotherapy for advanced disease.

## Figures and Tables

**Table 1:** Patient Characteristics.

		*N*	(%)
Total		51	(100%)
Sex	Male	46	(90.2%)
	Female	5	(9.8%)
Age at diagnosis, *years*	Median (range)	70	(47–85)
Symptoms at diagnosis		38	(90.5%)
ECOG PS	0	22	(45.8%)
	1–2	26	(54.2%)
Stage at diagnosis	I-III	38	(74.5%)
	IV	13	(25.5%)
Histology subtype	NEC	41	(80.4%)
	MANEC	10	(19.6%)
Perioperative chemotherapy ^+^		11	(29.7%)
Primary lesion resection		46	(90.2%)
Locally or metastatic residual resection after surgery		14	(27.5%)
Disease relapse after surgery		26	(70.3%) *
First-line chemotherapy for advanced disease		28	(54.9%)

^+^ Includes neoadjuvant and adjuvant chemotherapy; * out of the 37 patients with no residual disease after resection of primary; ECOG PS: Eastern cooperative oncology group performance status; NEC: neuroendocrine carcinoma; MANEC: mixed adenoneuroendocrine carcinoma.

**Table 2:** Cox proportional hazard models for the risk of death.

Factor	Univariate			Multivariate		
	HR	95%CI	*p*	HR	95%CI	*p*
Sex (Male)	0.99	0.30–3.27	0.980	-	-	-
Age	1.03	0.99–1.07	0.163	-	-	-
Symptoms at diagnosis	1.61	0.36–7.18	0.531	-	-	-
ECOG PS (PS 1)	2.26	0.99–5.17	0.053	ns	ns	ns
Stage (IV)	2.79	1.33–5.85	0.007	2.52	1.19–5.35	0.016
Histology subtype (NEC)	1.26	0.48–3.30	0.642	-	-	-
Ki67	1.02	0.99–1.06	0.147	-	-	-
Primary lesion resection	0.62	0.24–1.64	0.339	-	-	-

**Table 3:** Cox proportional hazard models for the risk of disease relapse or death.

Factor	Univariate			Multivariate		
	HR	95%CI	*p*	HR	95%CI	*p*
Sex (Male)	1.21	0.28–5.13	0.800	-	-	-
Age	1.01	0.97–1.05	0.750	-	-	-
Symptoms at diagnosis	1.25	0.29–5.43	0.770	-	-	-
ECOG PS (PS 1)	2.40	1.04–5.54	0.041	2.73	1.16–6.44	0.021
Nodal involvement	1.53	0.68–3.44	0.301	-	-	-
Histology subtype (NEC)	0.52	0.20–1.32	0.167	-	-	-
Ki67	1.02	0.99–1.05	0.285	-	-	-
Perioperative chemotherapy	0.47	0.20–0.14	0.096	0.42	0.17–1.05	0.065

**Table 4:** Cox proportional hazard models for the risk of progression or death.

Factor	Univariate			Multivariate		
	HR	95%CI	*p*	HR	95%CI	*p*
Sex (Male)	0.83	0.24–2.85	0.770	-	-	-
Age	1.06	0.99–1.12	0.063	1.06	1.00–1.11	0.036
ECOG PS (PS 1)	1.46	0.59–3.62	0.420	-	-	-
Surgery of primary lesion	0.68	0.23–1.99	0.485	-	-	-
Stage at diagnosis (stage IV)	1.07	0.47–2.42	0.874	-	-	-
Previous radical surgery	1.09	0.48–2.45	0.844	-	-	-
Histology subtype (NEC)	1.25	0.42–3.67	0.691	-	-	-
Carboplatin-etoposide chemotherapy	0.47	0.20–1.08	0.076	0.39	0.16–0.96	0.040

## Supplementary Materials

The following are available online at https://www.mdpi.com/2077-0383/9/5/1351/s1.
